# Neuroprotection Devices in Cardiac Catheterization Laboratories: Does It Sufficiently Protect Our Patients?

**DOI:** 10.3390/medicina61020305

**Published:** 2025-02-10

**Authors:** Clement Tan, Mark Daniel Higgins, Vaikunthan Thanabalasingam, Chaminda Sella Kapu, Zhihua Zhang

**Affiliations:** 1 Department of Cardiology, Mackay Base Hospital, Mackay, QLD 4740, Australiavaikunthan84@gmail.com (V.T.); chaminda.sellakapu@health.qld.gov.au (C.S.K.);; 2 Division of Medicine, Cairns Hospital, Cairns, QLD 4870, Australia; 3 The Prince Charles Hospital, Chermside, QLD 4032, Australia; 4 College of Medicine and Dentistry, James Cook University, Townsville, QLD 4814, Australia

**Keywords:** cardiac catheterization laboratories, electrophysiology laboratories, cerebral protection devices, cardiac embolic protection devices, neuroprotection devices, strokes, cerebral embolic lesions, patient safety, cost-effectiveness

## Abstract

Stroke is a devastating complication of cardiovascular interventions. Intraprocedural stroke is a well-documented and feared risk of cardiac percutaneous transcatheter procedures. If clinically significant strokes are absent, silent strokes remain the next in line to pose large concerns related to future cognitive decline, stroke risk, and overall increased morbidity and mortality. Cerebral protection devices (CPD) developed overtime aim to neutralize this risk through either a capture-based filter or a deflector mechanism. Many CPDs exist currently, each one unique, with varying degrees of evidence. The adoption of CPDs has allowed cardiac percutaneous transcatheter procedures to be carried out in patients with high thromboembolic risks who may have historically been discommended. Though skewed towards certain devices and transcatheter procedures, a large body of evidence is still present across other devices and procedures. This review will discuss clinical importance and respective stroke rates, updated evidence surrounding CPDs, differing opinions across types of CPDs, cost benefits, and what lies ahead for CPDs within the realm of procedures undertaken in cardiac catheterization laboratories.

## 1. Introduction

Cardiac catheterization laboratories (CCLs), commonly known as cath labs, have been increasingly used over recent years for percutaneous transcatheter procedures. F. Mason Sones Jr was the pioneering cardiologist who discovered coronary angiography by accident in 1958 at the Cleveland Clinic; his work has paved the way for the development of the field of interventional cardiology [1,2]. The establishment of percutaneous transcatheter procedures has omitted the need for thoracotomy and, in turn, allowed for the treatment of many patients who may be deemed poor thoracotomy candidates due to other comorbidities [3,4,5]. Since then, rapid evolvement has made way for more advanced technologies and improvements in adjunct pharmacology, operating scopes, and techniques, which allow these CCL procedures to be carried out at a steadily safer state with improved periprocedural outcomes [3,6].

More than a million people receive cardiac catheterization procedures year after year in the United States [6,7]. The indications for cardiac catheterization are plenty: they range mainly from diagnostic and therapeutic percutaneous coronary interventions (PCIs) to measuring haemodynamics in the right/left side of heart, evaluating left ventricular (LV) function, and assessing electrophysiological changes, valvular heart diseases, pericardial- and myocardial-originated diseases, congenital heart diseases, and heart failure, leading to improved life expectancy, quality of life, and overall functional status [8]. At present, approximately 90% of all procedures performed in CCLs across the world are typically PCIs, transcatheter aortic valve implantation/repair (TAVI or TAVR), left atrial appendage closure/occlusion (LAAC/LAAO), and arrhythmia ablations [1,2].

The 2021 Society for Cardiovascular Angiography and Interventions (SCAI) Expert Consensus Statement is a comprehensive document that covers the current best practices within CCLs. It provides key evidence-based information for issues regarding pre-procedure, intra-procedure, and post-procedure practices, aimed for use as guidance for the consistent delivery of high-quality care [7]. With the increasing use of cardiac catheterization procedures comes an increased interest in post cardiac catheterization complications. Like all medical procedures, the procedures in CCLs are not risk free. CCL procedures typically involve the use of arterial or intracardiac routes for access, which carry high risks in and of itself, hence showcasing the need to consider all forms of complications [9]. Complication rates are typically dependent on individualized factors such as demographics, vascular anatomy, comorbidities, acuity of clinical presentation, the procedure being performed, and operator experience [10]. The current 2021 SCAI Expert Consensus Statement recommends the use of risk scores to estimate peri-procedural PCI complications, though in clinical practice, risk scores are not typically used [1,7]. Well known risks scores such as STS and Euroscore have been used for years in measuring the likelihood of complications post coronary artery bypass graft (CABG). For example, the recommendation from the 2021 SCAI Expert Consensus Statement and its previous edition recommends the SCAI PCI Risk Calculator application, which uses the BMC2 calculator to assess risks of in-hospital mortality, transfusion, and contrast-induced nephropathy [7,11,12]. Another tool developed based on data from the National Cardiovascular Data Registry (NCDR), the Massachusetts Data Analysis Center (Mass-DAC), and DELTA models predicts risks such as mortality, femoral vascular injury, bleeding, dialysis, contrast-indued nephropathy, one-year target vessel revascularization, and 30-day readmission [12,13,14]. The risk can also be calculated using scores that consider individualized differences in coronary artery anatomy, such as the clinical SYNTAX score (CSS) or the New Risk Stratification score (NERS), which have had studies showing their high predictive ability in peri-procedural complications, death included [15].

When it comes to neurological complications, these complications, although rare, are some of the most dreadful due to the significant negative impact on key resource usage and on patients’ mortality and morbidity—increasing the risk of the development of dementia, future cerebrovascular events, and cognitive decline [6,7,10,16]. The risk of neurological events, like strokes or cerebral infarctions, can be influenced by some patient factors, such as age, gender, and co-morbidities, or even through mechanical causes from equipment used in CCLs [10,15]. The multiple equipment exchanges within the aortic root and long procedural times are also attributable factors [10]. For instance, the percutaneous transcatheters used in CCLs currently have recommended activated clotting times (ACT) ranging from 300 s to 350 s with the risk of thromboembolic neurological complications increasing as the duration of the procedure increases [17]. The guidewires used are also known to have a thrombogenic nature. Studies conducted on patient’s filters used in their respective percutaneous transcatheter procedures have found a myriad of different organic to inorganic tissue—arterial wall tissue, calcified tissue, necrotic core tissue of unknown origins, and inorganic material from hydrophilic polymer coatings of guidewires and catheters—all of which are clinically significant materials that pose as distal embolic risks leading to periprocedural cerebrovascular events [18,19,20].

The current 2021 SCAI Expert Consensus Statement has minimal information pertaining to neurological complications [7,21]. Neuroprotection devices or cerebral protection devices (CPDs) are temporary physical barriers with stability and filter capabilities that can be introduced through access sites of radial and femoral arteries during transcatheter procedures to provide a barricade at the ostium of aortic branches within the aortic arch. CPDs were all designed with the hope of being able to be used for most, if not all, percutaneous transcatheter procedures with a sole goal of reducing cardioembolic complications. The availability of studies evaluating CPD efficacy in CCLs are scarce. In general, most of the literature on CPDs are limited to TAVIs at present;where most focus on these devices’ safety, efficacy, incidence of subclinical magnetic resonance imaging (MRI) brain lesions, reduction in major adverse cardiac events (MACE), and overall mortality rates [22,23,24,25,26]. Thus, the aim of this present review would be to delve further into the field of CPDs, through discussing stroke incidences, highlighting available CPDs, discussing key clinical evidence surrounding a range of these devices and procedures, and outlining the growing recognition of CPDs in an array of percutaneous transcatheter procedures globally. A discussion of the cost-effectiveness and future of these devices will be conducted to help broaden the cruciality of their global adoption.

## 2. Cerebrovascular Events Within Cardiac Catheterization Laboratories

In present times, the high safety profile and general low complication rates associated with percutaneous transcatheter procedures have made them a desired method of management across clinicians. Nevertheless, their widespread application across the current aging population can carry a higher risk for periprocedural complications like cerebrovascular events [10,27]. These events can happen across procedures performed in CCLs, including mitral valve interventions, left atrial appendage occlusions, PCIs, and catheter ablations. Apart from clinically overt cerebrovascular events, silent strokes can also occur, sometimes known as cerebral embolic lesions (CELs), after these procedures—where patients may not present with focal neurological deficits but would have MRI findings suggestive of these silent brain lesions [28]. The predictors of periprocedural strokes, apart from age, are arterial hypertension, diabetes mellitus, the use of glycoprotein IIb/IIIa inhibitors, a history of stroke, renal failure, the use of an intra-aortic balloon pump, congestive heart failure, interventions at bypass grafts, and whether these procedures were being performed in emergent situations [6,27].

### 2.1. Transaortic Valve Replacement

From a patient’s perspective, stroke represents the most feared complication of TAVI [29]. Risk factors of TAVI are plentiful but can be split into mechanical, pharmacological, hemodynamic, and miscellaneous factors. Mechanical factors include fragments and debris from vessel walls and myocardium, endothelial injury, catheter manipulation, the use of balloon aortic valvuloplasty, and valve position and deployment [30]. The pharmacological factors are prothrombotic or anti-thrombotic states, including the use of anticoagulants. The hemodynamics intraprocedurally revolve around whether sluggish blood pressure was sustained, the rate and frequency of ventricular pacing, and the presence of hemodynamic instability [3,28]. The miscellaneous factors include periprocedural atrial arrhythmias like fibrillation or flutters, air emboli, and changes in blood flow dynamics in the neosinuses [31].

The Valve Academic Research Consortium (VARC) has established a classification for post-TAVI strokes into three separate categories; firstly, periprocedural stroke, occurring within 30 days of the procedure, which is further divided into acute stroke (within 24 h) and subacute stroke (between 24 h and 30 days); secondly, early stroke, which happens more than 30 days but within 1 year after the procedure; and lastly, late stroke, which occurs more than 1 year after the procedure [32,33]. Generally, stroke rates related to TAVI remain at 2–4%, with no significant changes at present [34,35]. The highest risk of stroke is usually within the first 48 h post-TAVI [36,37]. A meta-analysis performed of 10,037 patients across multiple studies globally found acute stroke rates at 1.5%, subacute strokes at 3.3%, and early strokes, divided across strokes at six and under twelve months at 4.3% and 5.2%, respectively [38]. Rates of late strokes specifically are scarce across the literature; however, a major TAVI registry from Switzerland found incidences of strokes were 4.3% at 1 year and 7.8% at 5 years [36]. Furthermore, the detection of clinical strokes and CELs post-TAVI is highly individualized depending on hospital protocols and the intensity of the neurological examination and imaging modality used [31]. The stroke rates in TAVI trials were dependent on the quality of neurological assessment, with higher rates detected when a neurologist conducts the evaluation. For instance, in the SENTINEL US IDE trial, the stroke rate was 9.1% in the control arm, compared to 1.8% in the FORWARD registry [39,40]. The 30-day stroke rate of 0.6% in the PARTNER 3 trial might be due to the low-risk patient cohort, the unclear use of CPDs, and variability in neurological assessment quality, which could have led to the lower incidence [41]. A meta-analysis of 64 studies with 72,318 TAVI patients showed a median 30-day stroke rate of 4% [42]. It has been proposed, however, that a 72 h stroke rate might be much more reflective of TAVI-related strokes as the 30-day stroke rate could reflect the development of other conditions that could have caused the strokes e.g., atrial fibrillation [28]. An extensive matched analysis conducted on three trials found a 72 h stroke rate of 5.4%, further emphasizing the importance of being vigilant post-TAVI, allowing for early detection and management where indicated [43].

An observational study that used the Society of Thoracic Surgeons-American College of Cardiology Transcatheter Valve Therapy (STS/ACC TVT) Registry database, which included over 120,000 TAVIs, observed no significant reduction in stroke with Sentinel CPD use; however, it suggested that patients with a history of stroke and peripheral arterial disease could benefit from CPD use [44]. The same observational study was conducted again recently in a larger cohort and time range—in 414,649 TAVI patients over 5 years—of whom, 53 389 (12.9%) had the Sentinel CPD used. The findings were positive in this larger cohort, wherein a small reduction in stroke associated with death or discharge to a location other than home—used as a proxy for disabling stroke—was observed [37]. One propensity-matched analysis supported these findings of a significant reduction in in-hospital strokes with CPD use [33]. A 2021 meta-analysis failed to show any clinical benefit, with no significant reduction in stroke incidence, and no difference in lesion volume on MRI observed [24]. A more recent 2023 meta-analysis that included the Sentinel, Embrella, Embol-X of randomized controlled trials has shown the benefits of how CPDs reduce the risk of death or stroke [23,24,45]. Notably, these meta-analyses do not include the most recent and largest-scale PROTECTED TAVR trial, which will be discussed in a later section.

### 2.2. Percutaneous Coronary Interventions

Cerebral micro-embolism is the main mechanism of peri-procedural ischemic strokes occurring with PCIs. This finding has been supported historically by transcranial doppler studies performed during cardiac catheterization showing the dynamic lodgement of cerebral micro-emboli [46,47,48]. Air embolism, thrombus from PCI catheters, or aortic atheroma from catheter manipulation and position are all possible sources of these micro-emboli [10].

The incidence of periprocedural stroke found in PCI registries ranged from 0.18% to 0.44%, with some studies having an unclear breakdown of ischemic versus haemorrhagic strokes [49,50,51]. While a New York registry study of 76,903 PCIs over a year, with a study definition of cerebrovascular accidents as transient or permanent new focal neurologic deficit post-procedure and before discharge from hospital, found an incidence of 0.2% [6]. It was unclear whether these PCIs were emergent, elective, or diagnostic, and what form of treatments were received during these procedures. Older literature, including one PCI register, reported an average incidence of 0.07% of strokes for diagnostic PCIs alone [52,53]. Another review, meanwhile, reported the incidence of cerebrovascular accidents for diagnostic cardiac catheterizations at 0.05–0.1% and 0.18–0.44% for patients receiving treatment during PCIs [27]. For strokes occurring post PCI, a study found an incidence of 0.28% for strokes within 7 days and another at 0.4% for 30-day incidence of strokes [28,54]. Interestingly, despite the development in the field, stroke rates seemingly remain unchanged. However, silent strokes were found in much higher proportions at 3.3% to 34.7% of patients, suggesting that these events are much more common [55,56]. Regardless, the cautious use of intra-arterial thrombolysis and mechanical embolectomy are relatively safe approaches in the treatment of periprocedural ischemic strokes after PCIs [10,46].

### 2.3. Mitral Valve Interventions

Percutaneous mitral valve repairs typically occur in the context of mitral regurgitation (MR) and mitral stenosis (MS) patients. MR remains the most common in the United States, affecting 2% of the population, and rising to 9% in those over 75 years old [57]. It is classified into primary (degenerative MR) and secondary (functional MR) types. MR, if untreated, leads to poor outcomes, but early intervention with mitral valve repair or replacement can improve survival. Surgery is the main treatment, while transcatheter options are emerging for high-risk patients. The three main types of these interventions include a transcatheter edge-to-edge repair, percutaneous mitral annuloplasty (both direct and indirect) and transcatheter mitral valve replacement/repair [3,57]. The COAPT trial, which randomized patients to the transcatheter mitral-valve repair using the MitraClip device, plus medical therapy, or medical therapy alone, found stroke rates at 30 days were 0.7% [58]. Another similar trial involving the same device, the MITRA-FR trial, reported 1.4% of cerebrovascular events periprocedurally and the stroke rate at 1 year was 3.9% [59]. Another smaller-scale study found silent brain lesions in 85.7% of their cohort of 27 patients, all of which did not progress to have any significant impact on cognitive function [60]. A systematic review and meta-analysis involving ten large studies found stroke rates after transcatheter mitral valve repair at 3.2%, much lower than that of surgical mitral valve repairs and optimal medical management groups [61]. There are limited data surrounding strokes after the use of Cardioband due to it being a newer device, as opposed to MitraClip. One major multicentre European study that took place over the course of 3 years found that the use of Cardioband led to periprocedural stroke rates at 1.6% [62]. Overall, the strokes rate in mitral valve interventions are lower compared with TAVI.

### 2.4. Left Atrial Appendage Closure/Occlusion

Stroke prevention in atrial fibrillation has become an increasing area of interest due to the rising prevalence of the condition with age and its strong link to increased overall stroke risk, especially cardioembolic strokes [8]. Traditional management approaches focused on long-term anticoagulation with warfarin or newer anticoagulants. However, growing evidence on the importance of left atrial appendage (LAA) thrombus has led to the development of mechanical approaches for stroke prevention, including surgical and catheter-based techniques. Multiple devices for left atrial appendage occlusion (LAAO) are currently being used, with LAAO being the second most common procedure, after TAVI, where CPDs are used. The types of LAAO devices affect the feasibility of the procedure in the presence of LAA thrombus. The traditional Watchman device adopts an umbrella shape, which requires the delivery sheath to be advanced up to the ostial plane of the LAA, posing a greater risk of distal touching and thus the embolization of thrombi. However, the newer Watchman FLX has newer mechanistic features, such as having a short device length and depth, which reduces this risk [63]. In contrast, loop devices like the Amulet have a shorter length that allows for shallower device deployment, thus reducing contact with thrombi. However, regardless of device, the partial or complete retrieval and re-deployment of either would significantly increase the risk of thrombi dislodgement.

The two landmark trials conducted using solely the Watchman device were the PROTECT AF and PREVAIL trial, each of them recording periprocedural strokes at 1.1% and 0.4%, respectively, with the latter having an overall higher procedural success rate [64,65,66]. One other study in a centre in Italy that compared the efficacy of LAAO using the Watchman device versus novel oral anticoagulants recorded 3.1% of periprocedural strokes [67]. The last published NCDR LAAO in 2019 had a record of 38,151 LAAO procedures, in which there were 45 cases, or 0.12%, of periprocedural ischemic stroke [68]. Limited data exist on new silent strokes after LAAO. In one study, 32% of patients treated with the Watchman device developed these silent strokes, while a correlation was observed between LAA angiographies and silent strokes in patients using Amulet, Occlutech, or LAmbre devices, and 4.8% of those treated with the Amplatzer Cardiac Plug, Watchman, or Amulet developed silent strokes [69,70,71]. When it comes to CPD usage in LAAO, a systematic review on 58 cases with unspecified CPDs found stroke in one case, and two device-related thromboses [72]. Two other literature sources, using available registries with a total of 31 LAAOs procedures performed with the use of a Sentinel device, found no strokes or device-related thromboses [45,73]. One other single-centre study conducted in Italy had 14 LAAO cases using a Sentinel and seven cases using the Triguard 3 and it found two access-related complications related to the Sentinel device and one access-related complication with Triguard 3 use [74].

### 2.5. Catheter Ablation

Catheter ablation offers a viable solution for patients with recurrent arrhythmias, but it is not void of stroke risk, especially when these ablations are performed in the left side of the heart. Radiofrequency and cryoballoon ablation are the two most used ablation modalities. Regardless of modality, transcranial doppler monitoring studies have shown concerning significant microembolic signals during their use in electrophysiology laboratories. In ascending order, these signals were lowest in cryoballoon ablation, then irrigated followed by non-irrigated radiofrequency ablation—with the latter known to be more thrombogenic [75,76]. Certain radiofrequency ablation catheters such as multielectrode-phased catheters reduce the risk of thromboembolic events and thus strokes. The use of higher powers, 90 watts and over, applied with shorter durations of 4 s or under, has also been linked to higher CEL occurrences [77,78,79]. The use of pulsed field ablation has a larger safety profile and has proven to be safer for patients. Outcomes from the IMPULSE, PEFCAT I, and PEFCAT II studies supported these through the findings of only one CEL and one TIA [80].

Atrial fibrillation ablation is the most common arrhythmia currently in the field of ablation [81,82]. The first 24 h post ablation up to the 2-week mark represents a high-risk period. The rate of periprocedural strokes in atrial fibrillation ablation, specifically left atrial catheter ablation ranged from 0.1% to 0.8% across some studies, while the rate of silent strokes was found to be at 50% for left atrial catheter ablations [83,84,85,86]. With ventricular tachycardia, periprocedural strokes were recorded in 0.8% to 2.7% of procedures, with the presence of structural heart disease having a higher risk, while the rate of silent strokes was slightly higher than that of atrial fibrillation ablation at 58%, likely due to the less standardized approach used in ventricular tachycardia ablations [87,88,89]. Notably, MRI studies have reported the occurrences of cerebral microembolisms, which can occur through several mechanisms: air or thrombus entering via sheaths, coagulum formation on the catheter or over-delivered ablation lesions, and gas bubble formation during the procedure. It also recommends the effective management of sheaths, the continuation of pre-procedural anticoagulation, maintenance of ACT > 300 s, and consideration of delaying post ablation cardioversion may reduce these risks [18,90]. The type of ablation and catheter used also influences this risk. For gaseous microembolisms specifically, many studies have shown that the introduction of air into the left atrium through the septal sheath during the placement of a ring catheter is thought to be its primary source [91,92,93]. Intra-cardiac thrombi remain a strict contraindication for VT ablation due to the high risk of embolization during the procedure, especially in patients with ischemic heart disease, where left ventricular thrombi are commonly found. Thrombi are rarely seen in conditions like dilated cardiomyopathy. In attempts to reduce the embolic risk, strategies such as peri-procedure anticoagulation, intracardiac echocardiogram, irrigated ablation catheters, and selective retrograde-aortic access have been trialed and have had minimal success [94]. In a study attempting to evaluate embolic risk in left ventricular ablation, it was found that despite sufficient ACT of 300 s to 400 s, 58% of patients developed a total of 16 cerebral emboli. Furthermore, despite the use of retrograde-aortic access in some of these patients, 63% still developed at least one new embolic infarct [89]. These further support a place and a need for CPD use in catheter ablation procedures. Two small-scale studies were performed to investigate CPDs in VT ablation, where one investigated the Sentinel device in 11 patients with unspecified severity of coronary artery disease and reported device debris in all cases and no strokes or device-related complications [95]. The other study, with an identical objective, used a mixture of Sentinel and Triguard 3 in seven patients and found similar outcomes [96]. One other single-centre study in Italy exploring CPD use in electrophysiological procedures in patients with thrombi, where nine patients underwent VT ablation, found no complications with the Sentinel device, and one arterial pseudoaneurysm with the use of the Triguard 3 device [74].

## 3. Cerebral Protection Devices in the Present Times

CPDs are used to prevent debris and clots from reaching the brain during procedures, reducing stroke risk. Clots may be present before or develop during the procedure [8]. CPDs are typically inserted through radial or femoral arteries, but device positioning can be difficult, especially if there are preprocedural atherosclerotic plaques in the supra-aortic vessels or aortic arch within the vicinity [23,31]. In this instance, the use of CPDs can disrupt plaques and cause cerebral embolization even before the procedure begins. Hence, in patients with profound risk factors like smoking, diabetes, obesity, or kidney disease, sometimes a preprocedural computed tomography angiography (CTA) may be recommended to detect these issues or other issues such as vascular tortuosity or aneurysms that could hinder proper device deployment [30,31,97]. Overall, the effectiveness of CPDs can be determined by its ability to protect the aortic arch’s main branches, brachiocephalic, left common carotid, and left subclavian artery, without causing harm to these vessels or arch and the ability of clinicians to deploy the device without disrupting pre-existing plaque material and its intraprocedural stability [97]. In the ideal setting, the features of a CPD should be the following: low cost, safe, stable, effective in capturing and removing emboli of a range of sizes without impeding cerebral blood flow, usable for all anatomical variations, simple to use, deploy, and retrieve, does not impede the use of procedural delivery systems, optimally visible under fluoroscopy, and able to be used across a myriad of procedures [31,98]. CPDs can be classified into filter-based capture devices, which include full body capture and remove devices, and deflector systems. An overview of the following devices’ advantages and disadvantages can be found in Table 1. The studies included below in each section of the different devices are also elaborated further in Table 2.

### 3.1. Filter-Based Capture Devices

#### 3.1.1. Sentinel (Boston Scientific)

The Sentinel is the most widely used and studied CPD and received the European Conformité Européene (CE) mark in 2014 [28]. It consists of two polyurethane mesh filters (140 µm pores) within a dual filter basket, advanced through a 6 Fr delivery catheter from the right radial artery over a 0.014-inch guidewire. The filters are positioned in the brachiocephalic and left common carotid arteries prior to procedures such as TAVI and are removed afterward. The device is deployed into supra-aortic vessels, requiring prior measurement of vessel diameters via a CTA. The proximal filter (9–15 mm) is placed in the brachiocephalic artery, and the distal filter (6.5–10 mm) in the left common carotid artery, with a potential additional filter for the left vertebral artery to ensure full cerebral protection [28]. Deployment usually takes less than 10 min in 91% of cases, although it slightly increases fluoroscopy time. The device has a recorded 94.4% success rate of device positioning [45]. A few years after receiving the CE mark, it became Food and Drug Administration (FDA)-approved in 2017 for embolic protection during TAVI, showing a low-risk profile and high potential for stroke prevention [31,99]. However, post marketing surveillance by the FDA reported 43 complications between 2017 and 2019, though the overall incidence rate remains unclear due to unknown total device usage and a lack of data on user experience or errors [100]. Despite this, the device remains a standard for cerebral protection during TAVI and other interventions. Sentinel as a CPD remains the CPD with the highest amount of available evidence, with other evidence also available for use in LAAO and VT ablation [74]. Thus far, the Sentinel device has shown mixed results in terms of reducing ischemic lesions and clinical neurological events. Early studies and smaller non-landmark trials demonstrated that the device did reduce ischemic lesions—up to a 50% reduction in the number of new lesions and lesion volume as assessed by diffusion-weighted MRI (DW-MRI). However, these studies did not show a statistically significant reduction in overall clinical neurological events at follow-up [101,102].

In early studies like MISTRAL-C and CLEAN-TAVI, which occurred in 2016, the device demonstrated some benefits in reducing cerebral lesions and improving overall neurocognitive function [102,103]. In the MISTRAL-C trial, 65 patients were enrolled and randomized, the Sentinel device group had fewer new cerebral lesions (73% vs. 87%, *p* = 0.31) and a lower volume of brain lesions (95 mm^3^ vs. 197 mm^3^, *p* = 0.171). Moreover, there was a significant reduction in patients with more than 10 lesions (0% vs. 20%, *p* = 0.03) and less cognitive disability (4% vs. 27%, *p* = 0.017) in the device group [104]. Similarly, in the CLEAN-TAVI trial of 100 patients, the Sentinel device group had fewer new lesions in protected brain areas (4 vs. 10, *p* < 0.001) and throughout the brain (8 vs. 16, *p* = 0.002), along with smaller lesion volumes (466 mm^3^ vs. 800 mm^3^, *p* = 0.02) [22]. The 2017 SENTINEL trial was another randomized study to evaluate the Sentinel device, involving a larger cohort of 363 patients from multiple centers across Germany and the United States. This study randomly assigned patients to three groups: a safety group with the Sentinel device use only and two MRI groups (one with the CPD and one without). The results showed that the CPD successfully captured embolic debris in 99% of patients, but the primary efficacy endpoint, the volume of new brain lesions, was similar between the groups within the MRI cohort (102.8 mm^3^ vs. 178 mm^3^, *p* = 0.33). While the 30-day stroke rate was numerically lower in the CPD group (5.6% vs. 9.1%, *p* = 0.25), it did not reach statistical significance [39].

The latest and largest scale study to date, the 2022 PROTECTED TAVR study, involved 3000 patients, 1833 patients in the United States and 1167 patients outside the United States, randomly assigned to receive either this device or control treatment, the incidence of 72 h peri-procedural stroke was 2.3% in the CPD group compared with 2.9% in the control group, a difference of −0.6% (*p* = 0.30), failing to demonstrate a statistically significant advantage of CPDs. However, the rate of disabling strokes was lower in the CPD group (0.5% vs. 1.3%, *p* < 0.05), suggesting a potential benefit in reducing disabling strokes, despite the suboptimal findings in its use in relation to 72 h peri-procedural stroke incidence [103]. Notably, a post hoc analysis performed of the trial found a greater stroke reduction in the United States cohort as opposed to the outside United State cohort. This suggested that locality differences could have affected patient characteristics and/or TAVI practices thus contributing to the subpar findings in the CPD as opposed to the control group [105]. Overall, the findings from Sentinel device studies suggest it to be a safe and effective device in capturing embolic material in most patients; however, its impact on reducing disabling strokes and cerebral damage and improving clinical outcomes, although promising, would require further studies to ascertain. Of note, a prospective large scale meta-analysis of the results of the completed 2022 PROTECTED TAVR study and the similar larger scale ongoing British Heart Foundation (BHF) PROTECT-TAVI study, which would combine a total of more than 10,000 TAVIs, is planned and would potentially provide an objective standpoint for the use of the Sentinel CPD in TAVIs [31,105].

#### 3.1.2. Emblok Embolic Protection (Innovative Cardiovascular Solutions)

The Emblok Embolic Protection System (EPS) is an investigational device designed to protect the brain and peripheral vasculature during TAVI or other left-sided heart procedures through a full circumferential coverage of the aortic arch. It consists of a 125 µm pore-sized nitinol filter integrated with a radiopaque pigtail catheter, which can be advanced through a single femoral puncture. The system accommodates aortic anatomies up to 35 mm in diameter [99]. In a first-in-human pilot non-randomized prospective study, the Emblok EPS was successfully placed in all 20 patients undergoing TAVI, with no procedural-related strokes at 30 days. Although debris was captured in 90% of patients, new CELs were detected in 95% of the cases [45,106]. The study concluded that the device was feasible and safe, though larger studies are needed to confirm its clinical benefits. A larger ongoing randomized trial, EMBLOK EPS trial (https://clinicaltrials.gov/study/NCT05295628 (accessed on 20 December 2024) Identifier: NCT05295628), with over 500 patients recruited, is currently underway for assessing the safety and effectiveness of the Emblok system, comparing it with other CPDs that have been in use [45,107].

#### 3.1.3. Wirion (Allium Medical)

The Wirion, an embolic protection device from Allium Medical, is used in carotid artery stenting and lower extremity transcatheter procedures to reduce embolic event risks. It features a filter basket made of self-expanding nitinol and a nylon membrane with 100 µm pores, along with a rapid exchange delivery catheter with a 1.1 mm crossing profile. The device is suitable for vessels ranging from 3.5 to 6.0 mm and works through delivery on 0.014 inch guidewires and through 6 Fr or larger delivery catheters [102]. However, it only protects one vessel at a time, making it unsuitable for high cardioembolism-risk procedures unless combined with other devices. As a result, there has been a study where its use was combined with the Sentinel device to provide comprehensive cerebral protection, especially during TAVI, safeguarding the left vertebral artery. While the Wirion was recalled for safety concerns, alternatives have been proposed for similar protection [108]. The WISE study (Wirion Study Europe) has been the only study thus far that has assessed the Wirion in 120 high-surgical-risk patients undergoing carotid artery stenting. The study showed lower complication rates compared with historical controls: mortality (0% vs. 1.7%), stroke (2.5% vs. 4.6%), and myocardial infarction (0.8% vs. 1.5%). The procedural and clinical success rates were 98.3% and 96.6%, thus the Wirion was found to be safe, effective, and associated with fewer complications in high-risk patients [102,108].

#### 3.1.4. Emboliner (Emboline)

The Emboliner total full-body embolic protection catheter, works as a capture-based filter CPD that extends across entire aortic arch with goal of complete cerebral and peripheral protection. The device has a mesh pore size of 150 μm, larger than other capture-based filter CPDs. Deployment of the device is through a contralateral access side using a femoral approach and a 9 Fr delivery catheter [28,74]. Currently being assessed in the SafePass 2 Trial, its feasibility was highlighted in the preliminary results presented at the Transcatheter Cardiovascular Therapeutics (TCT) symposium in 2019. The study demonstrated the device’s strong safety and technical performance with no adverse events at 30 days and a 100% procedural success rate. This represents the first quantitative data on the total amount of debris produced during TAVI. The findings suggest that, unlike partial protection devices, the Emboliner offers a significant improvement by providing more complete protection [109]. A pivotal trial that is underway known as the PROTECT H2H (Protect the Head to Head) trial (https://clinicaltrials.gov/study/NCT05684146 (accessed on 22 December 2024) Identifier: NCT05684146), is an international, prospective, randomized, two-arm study with the aim of assessing the Emboliner CPD compared with the control Sentinel CPD group in TAVI procedures for MACE. Recruitment for this study has already begun as of the first quarter of 2023 and will likely go through to the second quarter of 2024. Some early feasibility results acquired for the secondary efficacy endpoint showed efficiency of the Emboliner with all forms of TAVI systems and the successful capture of all debris larger than 150 μm [110].

#### 3.1.5. Captis (Filterlex Medical)

Filterlex Medical Ltd., Caesarea, Israel, introduced the Captis Embolic Protection System, with a design goal like that of the Emboliner in that it provides full-body embolic protection. Currently still undergoing development, Captis would use an ipsilateral transfemoral access and would be positioned in the aortic arch and descending aorta to safeguard both the brain and the peripheries from embolic events. The device would carry a mesh pore size of approximately 115 μm. The system would feature a filter-covered collapsible frame with filter pockets that capture embolic particles, preventing them from reaching critical areas like the renal system and aortic arch [28]. In a prospective, single-arm, non-randomized, first-in-human study known as the Captis study, 20 TAVI patients demonstrated 100% technical success with the device, showing effective deployment and retrieval without complications or interference with the TAVI procedure. Though results were only available for 11 of the 20 initial participants, there were no cerebrovascular events reported at 30 days, but one vascular complication at 30 days [31].

#### 3.1.6. Embol-X (Edwards Lifesciences)

The Emobl-X CPD is an aortic filter device providing full-body embolic protection. It was initially designed for cardiac surgeries with primary use in cardiopulmonary bypass through an aortic arterial cannula. The deployment of the self-expanding device is at the site of the ascending aortic arch through a 14 Fr sheath which consists of a heparin-coated, 120 μm polyester mesh in a flexible nitinol frame. It is available in five different sizes covering all aortic diameters from 22 to 40 mm. Though off-label, the device has been used in TAVIs for the same degree of embolic protection, despite its atypical direct access to the ascending aortic arch. When used in TAVIs, the Embol-X is instead deployed through a short 17 Fr sheath [102]. The initial reports of the Embol-X device used in TAVI involved three case series, showing limited information but had positive technical success and safety. In a prospective, single-blinded, randomized trial with 30 patients in Germany, of whom, 14 patients with TAVIs with Embol-X and 16 control group patients with TAVIs without Embol-X reported a 100% device deployment success. The post-procedure DW-MRI showed new brain lesions with restricted diffusion in 69% of the control group and 50% in the Embol-X group. The Embol-X group had smaller lesion volumes, with a significant reduction in the middle cerebral artery area (33 ± 29 mm^3^ vs. 76 ± 67 mm^3^, *p* = 0.04) [102,111,112].

#### 3.1.7. FLOWer (AorticLab)

Formerly known as the Embrace filter, FLOWer is a full-body embolic protection device designed to safeguard both cerebral and peripheral vessels during procedures, especially TAVI, which it has been primarily designed for. It consists of a cylindrical mesh filter with 70 μm pore size mounted on a frame that fits the aortic arch, covering all cerebral branch vessels and descending aorta. The device is available in three sizes and is intended to capture and remove debris created during TAVI. It uses a 12 Fr delivery system through the contralateral femoral, which includes a 5 Fr pigtail and a port that is compatible with all TAVI delivery systems. In an in vitro test reported by AorticLab, the device captured 99% of particles larger than 150 μm at the aortic arch (for cerebral protection) and 84% in the downstream circulation (systemic protection), with a calculated pressure drop of 6.6 mmHg at a cardiac output of 4.5 L/min [31,108].

The NAUTILUS trial, a single arm, prospective, multicentre trial, enrolled 75 patients undergoing TAVI. The preliminary findings from the study reported a 100% device deployment success. A 30-day stroke was reported in three patients. The median total new lesion volume at 2–5 days was of approximately 500 mm^3^ in 32 patients in the device arm and of approximately 1450 mm^3^ in four patients in the control group. Debris collected in 15 patients were recorded, which had an average collected particles of 420 each, with around 66% being particles ≤150 μm in size. The full findings of this trial have not been reported [31]. Of note, the FLOWer device has recently received a CE certification with the company planning a European postmarket clinical study and with the goal of a clinical study to obtain United States FDA certification.

### 3.2. Deflector Devices

#### 3.2.1. Embrella (Edwards Lifesciences)

The Embrella device was designed to deflect embolic material during TAVI. It later became approved for use in Europe in 2010. It is inserted via the right radial or brachial approach using a 6 Fr sheath. The device features an umbrella-like structure, hence its name, with a nitinol frame and two heparin-coated polyurethane membranes, with a pore size of 100 µm. Positioned through the greater curvature of the aorta, it provides partial protection to the supra-aortic vessels, excluding the left subclavian artery [28,31].

In the PROTAVI-C pilot trial, which included 52 patients, 41 with the device and 11 without, the device did not prevent cerebral emboli during TAVI. There was a 100% success rate in device deployment. While both groups developed CELs at 1 week, the device group had lower incidences of these. These CELs in both arms were all noted to be absent at the 30-day mark. Additionally, the device was associated with a significantly higher rate of heparin-induced thrombocytopenia (HIT) compared with the control group. Despite the increase in HIT, there were no associated cognitive or neurological impairments in the patients using the device. Overall, the Embrella did not prevent the occurrence of cerebral microemboli and it was dangerously linked to high incidences of HIT. Hence, the device was subsequently discontinued, and is no longer in development [31,74,113].

#### 3.2.2. Triguard 3 (Venus Medtech)

The TriGuard system is the second most studied CPD after the Sentinel device. It was developed to provide cerebral protection during TAVI. It consists of a deflection filter that covers all three supra-aortic vessels, including the left subclavian artery, and can be left in the aortic arch for a small number of days. The latest version, TriGuard 3, features a self-expanding nitinol frame with an ultra-thin heparin-coated polymer mesh, designed to deflect particles larger than 140 µm. It is inserted via a 9 Fr femoral sheath, and its over-the-wire design allows for concurrent use of a 6 Fr pigtail catheter [45,74].

The TriGuard system has been evaluated in several clinical trials, with mixed results. The DEFLECT I and DEFLECT II trials, which were single-arm studies, showed no significant reduction in the number of CVAs compared with historical controls. The DEFLECT III trial, a multicentre, randomized study, did not find significant differences in in-hospital procedural safety outcomes between patients with the TriGuard device and the control group [31,114]. The REFLECT I trial, a randomized controlled trial involving 258 patients, was suspended before completion due to safety concerns. It found no significant differences in the primary efficacy endpoint, which included mortality, stroke, cognitive decline, and cerebral ischemic lesions. The latest REFLECT II trial assessed the device in 220 patients undergoing TAVI. It met its primary safety endpoint, showing a significant reduction in major adverse events. However, the primary efficacy endpoint, which included stroke, cognitive decline, and cerebral ischemic lesions, was not met. The device achieved complete coverage of all three cerebral vessels in 59.7% of cases, but device-related complications occurred in 9.6% of patients. Hence, TriGuard 3 demonstrated a safety benefit but not a significant improvement in clinical efficacy [22,28,31]. In recent years, the TriGuard system has been increasingly used in LAAO and ventricular tachycardia ablation procedures, showing encouraging results. However, while it offers significant safety advantages in terms of stroke prevention, its efficacy in reducing cerebral lesions and improving clinical outcomes remains inconclusive [74,96].

#### 3.2.3. ProtEmbo (Protembis)

The ProtEmbo CPD received European CE mark approval in 2014. This CPD covers all three supra-aortic vessels and is designed for delivery via left radial access with a low-profile design. The proximal part of the device is anchored in the left subclavian artery ensuring coverage of the left vertebral artery, and when the filter is deployed, the rest of the frame sits in the roof of the aortic arch covering the ostia of the left common carotid and brachiocephalic arteries thus filtering all the blood flowing to the brain. It features a heparin-coated mesh with the smallest pore size of 60 µm among other available CPDs, potentially offering extra protection against smaller sized debris [45,115].

The PROTEMBO C trial evaluated the safety and performance of the device in TAVI patients. The device met its primary safety and performance endpoints, showing smaller brain lesion volumes on DW-MRI compared with historical data, with no large lesions (>150 mm^3^) [116]. The PROTEMBO SF trial, much like the PROTEMBO C trial, is a prospective, observational, multicentre study assessing the safety and feasibility of the ProtEmbo use in patients with severe symptomatic native aortic valve stenosis undergoing TAVI [115]. Despite only having five subjects enrolled and not fully reported, the preliminary findings demonstrated a 50% reduction in the number of new lesions compared with previous cohorts, and an 87% reduction in new lesions with Protembo use in TAVI at 3 and 30 days [31]. The PROTEMBO investigational device exemption (IDE) trial (https://clinicaltrials.gov/study/NCT05873816 (accessed 22 Decmber 2024) Identifier: NCT05873816) conducted by Protembis is a multi-centre, randomized study of 500 patients to assess the safety and efficacy of the ProtEmbo CPD against the Sentinel and no CPD use is currently underway at its recruitment phase.

#### 3.2.4. Point-Guard (Transverse Medical)

The Point-Guard system is designed to cover all the major arch vessels, offering protection against embolic debris during TAVI or other left-sided heart procedures. It consists of a flexible nitinol frame with a filter mesh encircling its perimeter and a supporting extension at the distal end. The device’s isolation zone is intended to stabilize it dynamically during positioning, helping prevent migration and decoupling causing failure of device [45,108].

The CENTER trial is a non-randomized trial that included four subjects undergoing TAVI, and had findings presented but not published yet. There was, however, evidence of debris capture in all subjects. All subjects also experienced non-device-related adverse events, although the specifics of it are not known yet [31]. A randomized trial, the GUARDIAN trial, to be conducted by Transverse Medical, has not yet been registered but may occur in the near future for the purpose of assessing the safety and efficacy of the Point-Guard CPD [31].

**Table 1:** Summary of the advantages and disadvantages of different cerebral protection devices.

Classification	Device	Advantages	Disadvantages
Filter based devices	Sentinel [22,28,45]	▪ Largest body of evidence—especially for TAVIs, and some LAAO and VT ablation ▪ Becoming a standard for use during TAVIs ▪ CE and FDA approved ▪ Successful deployment rate of 94.4% ▪ Has addition filter for protection of left vertebral artery ▪ Significant reduction in CELs and stroke rates	▪ Partial protection—does not cover for peripheral vessels ▪ Limited evidence in other transcatheter procedures ▪ Large number of promising findings but not all statistically significant
	Emblok [45,106]	▪ Success deployment rate of 100% ▪ Accommodates larger aortic diameters ▪ Full circumferential coverage of aortic arch and peripheral vasculature ▪ Randomized clinical trial underway ▪ No periprocedural stroke detected	▪ Limited concrete evidence at present ▪ Large number of CELs detected
	Wirion [102,108]	▪ Successful deployment rate of 98.3% ▪ Successful reduction in mortality, stroke, and myocardial infarction when used in carotid artery stenting	▪ Single vessel protection at a time ▪ Mainly used in carotid artery and lower extremity transcatheter procedures ▪ Not suitable for all transcatheter procedures, e.g., those with high embolic risks ▪ Evidence for use in conjunction with Sentinel for other transcatheter procedures is pending
	Emboliner [109,110]	▪ Full coverage of aortic arch and peripheral vasculature ▪ Successful deployment rate of 100% ▪ No strokes detected at 30 days ▪ Randomized clinical trial underway with some promising early results	▪ Limited concrete evidence at present ▪ Upcoming RCT only compares it to Sentinel, not other devices
	Captis [31]	▪ Full coverage of aortic arch and peripheral vasculature ▪ Successful deployment rate of 100% ▪ No strokes detected at 30 days	▪ In developmental stages ▪ Limited concrete evidence at present ▪ Single vascular complication associated with device use
	Embol-X [102,111,112]	▪ Full coverage of aortic arch and peripheral vasculature ▪ Successful deployment rate of 100% ▪ Lesser and smaller CELs detected	▪ Limited concrete evidence at present ▪ CELs detected in half of cases
	FLOWer [31]	▪ CE approved ▪ Full coverage of aortic arch and peripheral vasculature ▪ Successful deployment rate of 100% ▪ Large amounts of debris collected of various sizes ▪ Smaller CELs when used	▪ Small number of strokes at 30 days ▪ More CELs detected when used
Deflector	Embrella [28,74,113]	▪ Successful deployment rate of 100% ▪ No CELs detected at 30 days	▪ Partial protection—does not cover left subclavian artery and peripheral vessels ▪ CELs detected when used—albeit present in both groups ▪ Increases risk of heparin induced thrombocytopenia
	TriGuard 3 [22,74,114]	▪ Second largest body of evidence ▪ Successful deployment rate of 100% ▪ Can be left in situ for some days ▪ Single non-randomized trial showing significant reduction in strokes ▪ Promising evidence for use in LAAO and VT ablation	▪ Partial protection—does not cover peripheral vessels ▪ Multiple trials showing no reduction in strokes or CELs ▪ Small number of device-related complications
	ProtEmbo [31,115]	▪ CE approved ▪ Lesser and smaller CELs when used ▪ Single small-scale trial with preliminary findings of significant reduction of periprocedural and 30 days strokes ▪ Large scale trial underway currently	▪ Partial protection—does not cover peripheral vessels ▪ Limited concrete evidence at present
	Point-Guard [31,108]	▪ Has an innate isolation zone to prevent migration while in situ ▪ Successful deployment rate of 100% ▪ Single small-scale trial demonstrated debris capture ▪ RCT underway	▪ Partial protection—does not cover peripheral vessels

TAVI/R = Transcatheter aortic valve implantation/repair; LAAO/C = Left atrial appendage occlusion/closure; VT = Ventricular tachycardia; CE = Conformité Européene; FDA = Food and Drug Administration; CEL = Cerebral embolic lesion; RCT = Randomized controlled trial.

**Table 2:** Summary of various studies including limitations.

Device	Study	Year	Study Type	No. of Patients	Key Endpoint/s	Findings	Limitations
Sentinel	MISTRAL-C [104]	2016	Randomized controlled trial	65 (Device arm: 32; Control arm: 33)	Incidence of new brain lesions 5 to 7 days after TAVI, assessed by DW-MRI; neurocognition decline	▪ Strokes: 1 vs. 6 ▪ New brain lesion at 5 to 7 days: 73% vs. 87%, *p* = 0.31 ▪ Median total new lesion volume: 95 mm^3^ (10–257) vs. 197 mm^3^ (95–525) (*p* = 0.17); Median total lesion volume in protected areas: 0 mm^3^ (0–102) vs. 76 mm^3^ (40–221), *p* = 0.057 ▪ Absence of new lesions: 13% vs. 27%, *p* = 0.31 ▪ New neurocognitive decline: 4% vs. 27%, *p* = 0.017 ▪ Total lesion volume larger with self-expanding TAVI vs. ballon-expandable: 693 mm^3^ vs. 266 mm^3^, *p* = 0.067	The study had a small sample size and lacked sufficient power due to an unexpectedly higher follow-up MRI dropout rate. Despite randomization, patients without device use had significantly higher Society of Thoracic Surgeons risk assessment scores and more major vascular complications, though a large proportion of these patients did not complete MRI or neurocognitive follow-up, hence would have had unknown outcomes. The study focused only on the early postoperative period, i.e., 5 to 7 days, and the long-term impact would have been unclear. Especially vital for a time where these CPDs are starting to gain ground. Mistral-C did, however, set the trajectory for other larger trials for the Sentinel device.
	Clean-TAVI [22]	2016	Randomized controlled trial	100 (Device arm: 50; Control arm: 50)	Reduction in post-procedure brain lesions on DW-MRI relative to baseline at 2 days following TAVI	▪ Stroke incidence within 7 days: 10% vs. 10% (non-disabling form) ▪ New cerebral lesions: 98% ▪ Number of new lesions: 4 (3–7.25) vs. 10 (6.75–17), *p* < 0.001 ▪ New lesion volume: 242 mm^3^ (159–353) vs. 527 mm^3^ (364–830), *p* = 0.001	The study was only a single-center study that used a single type of TAVI device and a single experienced team of clinicians—limiting the generalizability of its findings to broader populations especially internationally, where other devices and procedural techniques are used. The study aimed for a 50% reduction in new brain lesions, but this seemingly arbitrary target has an uncertain clinical relevance. Neurological and neurocognitive outcomes were hypothesis-generating and lacked routine assessments by neurologists. The device’s limitation of not protecting the left vertebral circulation was discussed, though the focus of post-procedure brain lesions were on areas not typically supplied by the left vertebral artery. Additionally, the unblinded team could have introduced a degree of bias across the supposed randomization, though standard randomized control trial guidelines were followed throughout.
	Sentinel [39]	2017	Randomized controlled trial	363 (Device safety arm: 123; Device imaging arm: 121; Control arm: 119)	Safety: incidence of MACCE at 30 days: all death, all strokes (disabling and non-disabling), and acute kidney injury (AKI; stage 3) according to VARC-2 criteria Efficacy: reduction in median total new lesion volume in protected territories between both arms, assessed by DW-MRI at 2 to 7 days after TAVI	▪ Debris found within filters: 99% ▪ MACCE: 7.3% vs. 9.9%, *p* = 0.41 ▪ New lesion volume: 102.8 mm^3^ vs. 178 mm^3^; *p* = 0.33 ▪ 30 days stroke: 5.6% vs. 9.1%, *p* = 0.25	The study showcased safety and feasibility for the device. But it is known that the device did not protect the left vertebral artery, and findings of residual lesions in protected areas suggest that the device at the time of study could have been considered inadequate at capturing debris or that embolization continues after filter removal. MRI follow-up was missing in 25% of patients due to noncompliance and/or the need for pacemaker post-TAVIs—a known complication of TAVIs. Despite having a large population, some might consider the sample power insufficient to assess clinical, MRI, or neurocognitive outcomes.
	PROTECTED TAVR [103]	2022	Randomized controlled trial	3000 (Device arm: 1500; Control arm: 1500)	Clinical stroke within 72 h after TAVI or before discharge (whichever came first)	▪ Clinical stroke: CPD 2.3% vs. control 2.9%, *p* = 0.30 ▪ Disabling stroke: 0.5% vs. 1.3%, *p* < 0.05 ▪ Death: 0.5% vs. 0.3% ▪ TIA: 0.1% vs. 0.1%	The outcome data in this study were limited to a narrow set of endpoints with only short-term follow-up. Neurologists were not blinded to patients’ clinical histories and hospital records, which could have ultimately influenced stroke reporting. Additionally, despite randomization and a large patient pool, it remains unknown why investigators had a higher percentage of female patients in CPD group than the control group. The nature of the female gender itself is a known stroke risk factor and thus could have inflated the strokes rates slightly in this study. Lastly, the trial findings are specific to a specific Sentinel CPD device model investigated and may not apply to others.
Emblok	Emblok [106]	2020	Multicentre, prospective, non-randomized, first-in-human	20 patients submitted to TAVI with Emblok	Success and immediate cerebral embolic burden after TAVI (number and volume of new brain lesions assessed by DW-MRI at days 2 to 5 post-TAVI compared with baseline)	▪ Significant debris capture: 90% 30-day MACCE: 0% ▪ New ischemic defect post-procedural DW-MRI: 95% ▪ Median number of new lesions per patient: 10 (4.75–15.2) ▪ Total new lesion volume: 199.9 mm^3^ (83.9–447.5) ▪ Mean lesion volume per lesion: 42.5 mm^3^ (21.5–75.6)	This study aimed to assess the feasibility of the Emblok system rather than providing definitive evidence of its protective benefits, making it purely an exploratory study by definition and not powered to detect statistically significant effects on other endpoints, so the results should be viewed as hypothesis-generating. The use of a 1.5-T MRI scanner, rather than a 3-T scanner, may have led to an underestimation of the cerebral embolic burden since 3-T scanners are known for better resolution and overall image quality. Since the trial was not randomized without a control group, it cannot conclusively evaluate the device’s effectiveness in reducing cerebral embolic damage after TAVI. Additionally, the follow-up period was only limited to 30 days, so the long-term neurological and cognitive outcomes remain unknown.
Wirion	WISE [117]	2017	Multicentre, prospective, non-randomized, single-arm	120 patients submitted to carotid artery stenting with Wirion (Excludes 22 roll ins)	Freedom from stroke, myocardial infarction, and death within 30 days post-procedure	▪ Mortality: 0% vs. 1.7%, *p* = 0.21, ▪ Stroke: 2.5% vs. 4.6%, *p* = 0.18, ▪ MI: 0.8% vs. 1.5%, *p* = 0.50 ▪ Roll in cases had no MACCE	The study was not non-randomized but rather took an observational approach which would most definitely introduce selection bias, limiting the reliability and overall generalizability of the results. Furthermore, the procedure was carotid artery stenting instead of TAVIs or other transcather procedures which all carry gross anatomical differences, i.e., proximity to the brain. Historical data were also used from other CPDs instead of at the time of the study as a comparison to the study’s findings. In this study, a notable proportion of the subjects were healthier individuals in that they were not as symptomatic, had lower stroke risks, or had not had TIAs in the past but with notable carotid atherosclerosis.
Emboliner	SafePass 2 [31,109]	2019	Multicentre, prospective, non-randomized	31 patients submitted to TAVI with Emboliner	Incidence of 30-day MACCE (death, stroke, and stage 3 AKI) compared with a 12% historical performance goal	▪ MACCE: 6.5% posing 46% reduction compared to historical performance goal ▪ Debris capture: 100%	The study had several limitations impacting its conclusions. The small sample size of 31 patients hindered the detection of significant differences or rare adverse events, making it difficult to definitively assess the device’s efficacy. The lack of a control group in this single-arm study also limited the ability to attribute observed benefits solely to the device. Patient selection criteria introduced potential bias, possibly excluding those with complex anatomy or higher risks, which greatly limits the findings’ generalizability to the broader TAVI population. Additionally, the short follow-up period may have missed long-term neurological complications. As a first-in-human study, investigator bias could have influenced the interpretation of results and the overall device’s efficacy.
Captis	Captis [118]	2023	Two centres, prospective, single-arm, first-in-human	20 patients submitted to TAVI with Captis	Incidence of all MACCE at 72 h post-procedure	▪ MACCE: 0% at 30 days ▪ No increase of NIHSS score at follow-up. 1/11 patients had an increase in mRS score at 72 h and 2/10 patients at 30 days	The study was not appropriately designed to test efficacy; as the clinical outcomes cannot be simply concluded due to the small cohort, and the lack of post-procedural DW-MRIs. But the strong suggestions of its safety and efficacies were found in the meticulous pre- and postprocedural neurological assessment of the captured debris. The device was available in limited sizes, and patients with a complex aortic anatomy and/or descending aorta diameter smaller than 20 mm or larger than 27 mm were excluded. Thus, these study findings should be considered when interpreting the results. This study does, however, provide an encouraging trajectory for larger high risk cohort studies to investigate proper feasibility and safety proof of this device during TAVIs.
Embol-X	Embol-X [112]	2015	Randomized controlled trial	30 (Device arm: 14; control arm: 16)	Number of new lesions on DW-MRI and lesion size	▪ No strokes reported ▪ New lesion on DW-MRI: 57% vs. 69%, *p* = 0.70 ▪ Lesion size: 88 ± 60 mm^3^ vs. 168 ± 217 mm^3^, *p* = 0.27Lesion volumes in the supply region of the middle cerebral artery: 33 ± 29 mm^3^ vs. 76 ± 67 mm^3^, *p* = 0.04	The study was prematurely terminated for safety concerns; however, some important findings were available. Transcranial doppler was not used when TAVIs were performed. Though randomized, the findings should be interpreted and used with caution and would require upcoming larger scale study to corroborate these findings.
FLOWer	NAUTILUS [31]	2023	Multicentre, prospective, single-arm	75 (With 5 different TAVI models used—though not split equally amongst models)	Safety: incidence of 30-day MACCE (death, stroke, and stage 3 AKI) compared with a 14.3% historical performance goal Clinical benefit: DW-MRI at baseline and within 2–5 days after TAVI; neurocognitive protection assessed by NIHSS, MOCA and mRS at baseline, 2–7 days and 30 days after TAVI Performance: success and system usability; debris captured	▪ 3 (5.2%) strokes at 30 days (data from 58 patients) ▪ Median total new lesion volume at 2–5 days: device arm: around 500 mm^3^ (32 patients); control arm: around 1450 mm^3^ (4 patients) ▪ Debris collected in all cases, with an average total number of collected particles of 420 per patient, with around two-thirds being particles ≤150 μm in size (data from 15 patients)	The study was purely observational, with a small sample size that very much lacked sample power, hence precluding any in-depth comparison against a control group or detailed analysis related to other endpoints assessed in other similar studies of other CPDs. Although study was multicenter, it was limited across 2 European countries, which could lead to results not being generalizable internationally due to different norms in operator training across the globe.
Embrella	PROTAVI-C [113]	2014	Prospective, non-randomized trial	52 (Device arm: 41; Control arm: 11)	Periprocedural cerebral lesions assessed by DW-MRI	▪ 7 days DW-MRI new ischemic lesions: 100% vs. 100%; ▪ Median number of defects per patient: 8 (3–13) vs. 4 (2–8), *p* = 0.41 ▪ Lesion volume per lesion: 30 mm^3^ (20–50) vs. 50 mm^3^ (30–70), *p* = 0.003	The study was not randomized and had relatively small sample sizes despite a control group. No major differences were detected between control and CPD groups, but this should be used with caution. The small sample size of the control group would render insufficient statistical power to draw any meaningful conclusions. These results need to be confirmed by a larger, preferably randomized, study.
TriGuard 3	DEFLECT I [119]	2015	Multicentre, prospective, single-arm	37 patients submitted to TAVI with TriGuard	In-hospital device- or procedure-related cardiovascular mortality, major stroke disability, life-threatening bleeding, distal embolization, major vascular complications, or need for urgent cardiac surgery	▪ Primary outcome: 8.1% ▪ New cerebral is chaemic lesions on post-procedure DW-MRI: 82% ▪ Per-patient total lesion volume: 34% lower than historical cohorts (0.2 cm^3^ vs. 0.3 cm^3^)	The study was not randomized and was a comparatively small-scale study despite being the first study of the TriGuard device. Safety comparisons with unprotected TAVI must be taken with caution, due to the small-scale, lack of an active control, and how the procedural aspects of TAVIs have evolved since then. The study was powered to detect reductions in new lesions, but there were no controls involved that had similar DW-MRI investigations for detecting new lesions. The use of statistics from historical cohorts were from unprotected TAVIs, but the nature and risk profile of these unprotected TAVIs were not appropriately listed. There were follow-up challenges which included 24% loss to DW-MRI at 4 ± 2 days and 43% loss to complete cognitive and neuroimaging follow-up, reflecting difficulties that investigators can often face when elderly, multimorbid TAVI patients are included in sample pool. DW-MRI was not performed in two major stroke cases, potentially artificially downplaying true lesion volumes. Cognitive outcomes were assessed using the MoCA to detect subtle changes, but its ability to identify meaningful impairment in this population remains unvalidated against comprehensive neuropsychological testing.
	DEFLECT II [120]	2015	Multicentre, prospective, single-arm	15 patients submitted to TAVI with TriGuard	In-hospital MACCE occurrence, number and volume of new cerebral lesions on DW-MRI	▪ Primary outcome: 0% ▪ New cerebral ischaemic lesions on post-procedure DW-MRI: 78.8% vs. 88.5% ▪ Per-patient new lesion: 5.5 vs. 5.5 median, *p* = 0.96 ▪ Per-patient lesion volume: 98.9 vs. 129.4 median, *p* = 0.16	This study’s prospective and single-arm feasibility study nature accurately showcased the device performance success (67% achieved) with no MACCE occurrence. The study does possess a small sample size, lack of a control group, incomplete DWI data (only 8 patients), short follow-up period, and no long-term neurological or cognitive assessments which would render its findings limited for use. Additionally, the variability in valve types and the suboptimal device success rates would suggest a significant need for larger, preferably randomized, controlled trials to validate these findings and better understand the device’s efficacy and broader applicability.
	DEFLECT III [114]	2015	Randomized controlled trial	85 (Device arm: 46; Control arm: 39)	In-hospital procedural safety (death, stroke, life-threatening or disabling bleeding, stage 2/3 acute kidney injury, or major vascular complications) Efficacy: cerebral ischemic lesions on DW-MRI; neurocognitive deterioration	▪ Strokes within 72 h: 2.2% vs. 5.1% (*p* = 0.30) ▪ New brain lesions at 30 days: 73.1% vs. 88.5% ▪ Worsening NIHSS score from baseline with DW-MRI evidence of ischaemia: 3.1% vs. 15.4% (*p* = 0.160) ▪ New post-TAVI DW-MRI detected ischaemic lesions at 30-day follow-up: 11.5% vs. 9.1% (both mean single and maximum lesion volumes were 5.2 ± 17.9 vs. 3.3 ± 11.9 mm^3^; *p* = 0.78)	The study was performed across many centers; however, its relatively small sample size does limit the reliability of the results. Additionally, the study design is exploratory, with a stronger focus on safety and efficacy rather than long-term outcomes, leaving uncertainties beyond the 30 days mark. The assessment of cognitive function was limited to the MoCA and a delayed memory task, which are not fully validated in these contexts, and thus would not fully capture the broader spectrum of cognitive decline. Furthermore, since the study was conducted, there have been significant advances in TAVI technology and techniques that would influence the relevance of the findings to current practice.
	REFLECT I [121]	2021	Multicentre, prospective, single-blind, 2:1 randomized	258 (Device arm: 141; Control arm: 63; 54 roll ins)	Safety at 30 days: VARC-2, a composite of all-cause death, stroke, life-threatening or disabling bleeding, stage 2–3 AKI, coronary artery obstruction requiring intervention, major vascular complications, and valve-related dysfunction requiring repeat intervention Efficacy: a composite of (1) all-cause mortality or any stroke at 30 days, (2) NIHSS worsening from baseline to 2–5 days post-procedure or MoCA worsening (decrease of 3 points or more from baseline) at 30 days, and (3) total volume of cerebral ischaemic lesions detected by DW-MRI performed 2–5 days post-procedure	▪ Primary safety outcome was met compared with the performance goal (21.8% vs. 35%, *p* < 0.0001) ▪ Primary hierarchical efficacy endpoint was not met (mean efficacy score, i.e., higher the number meaning higher efficacy: −5.3 ± 99.8 vs. 11.8 ± 96.4, *p* = 0.31) ▪ Covert central nervous system injury was lower both in-hospital for device (46.1% vs. 60.3%, *p* = 0.0698) and at 5 days (61.7 vs. 76.2%, *p* = 0.054). ▪ NIHSS or MoCA worsening post-procedure or at 30 days: 18.1% vs. 13.3%, *p* = 0.5286 ▪ Total median cerebral lesion volume: 231 mm^3^ (56–631) vs. 243 mm^3^ (84–484), *p* = 0.9448	The trial was suspended early, limiting the sample size of the trial, but gave some promising results. Apparent differences in secondary endpoint rates should be interpreted with caution due to the small overall sample size, imbalanced randomization, and unrepresentatively low control group event rates. In addition, patient withdrawal from the study and losses in clinical and DW-MRI follow-up limit secondary efficacy comparisons, which are underpowered and should be considered hypothesis-generating.
	REFLECT II [122]	2020	Randomized controlled trial	220 (Device arm: 162 [41 roll-ins plus 121 randomised]; Control arm: 58)	Safety at 30 days: a composite of all-cause death, stroke, life-threatening or disabling bleeding, stage 2–3 AKI, coronary artery obstruction requiring intervention, major vascular complications, and valve-related dysfunction requiring repeat procedure Efficacy: a hierarchical composite of (1) all-cause mortality or any stroke at 30 days, (2) NIHSS worsening from baseline to 2–5 days post-procedure or MoCA worsening (decrease of 3 points or more from baseline) at 30 days, and (3) total volume of cerebral ischaemic lesions detected by DW-MRI performed 2–5 days post-procedure	▪ Strokes in hospital: 6.4% vs. 5.3% in hospital, *p* = 1.000 ▪ Safety endpoint: 15.9% vs. 7%, *p* = 0.11 ▪ Primary efficacy endpoint at 30 days (all-cause mortality, all stroke, NIHSS worsening, absence of DW-MRI lesions post-procedure, total volume of cerebral lesions by DW-MRI): 45.7% vs. 54.3%, *p* = 0.857 ▪ Median total new lesion volume at 2–5 days: device arm: 215.39 mm^3^; control arm: 188.09 mm^3^, *p* = 0.405 ▪ NIHSS scores at discharge: 14.1 vs. 7.6, *p* = 0.18, or at 30 days post-procedure: 7.8 vs. 3.6, *p* = 0.31	Trial was halted before completing the planned enrolment. As a result, the sample size was smaller, which may have led to differences in baseline characteristics. The study’s ability to achieve its pre-specified primary efficacy endpoint was slightly altered, hence the comparisons made should be considered exploratory and should be interpreted with caution. Moreover, the safety performance goal was based on data from earlier clinical trials, which might not accurately reflect current procedural changes and associated risks and should be considered within that context.
ProtEmbo	ProtEmbo SF [31,102]	2018	Multicentre, prospective, observational, intention-to-treat	4 patients submitted to TAVI with Protembo	Feasibility and safety	▪ Stroke at 30 days: 0% ▪ 50% reduction in new lesions compared with identical comparison subjects, and 87% reduction of new lesions for the protected vs. unprotected TAVI at 3 and 30 days ▪ No difference in MoCA at 30-day follow-up ▪ Minimal to no interaction with TAVI catheter types were reported	With a small sample size of 4, it would be difficult to draw any forms of conclusion from this study. There was also uncertainty surrounding the inclusion and exclusion criteria of patients. However, being a first-in-human trial, the findings do attempt to pave way for larger trials like the ProtEmbo C trial. The comparison to another subject may be wholly difficult to ascertain any forms of reliability, due to uncertainty around the study in outlining the comparison subjects used in this instance. Furthermore, only an abstract of study was published.
	ProtEmbo C [115]	2022	Multicentre, single-arm, intention-to-treat	41 (37 intention-to-treat cohort, of which 31 per-protocol cohort)	Safety: MACCE at 30 days, defined as a composite of all-cause mortality, all stroke, life-threatening or disabling bleeding, major vascular complications in the access vessels or aorta, or AKI (stage 2 or 3), all according to VARC-2. Performance: device success, ability to secure and stabilise the position of the device through procedure; and deflection of embolic material, defined by coverage of all 3 cerebral vessels without affecting flow	▪ MACCE at 30 days was 8.1% (21.3% vs. performance goal of 25%; *p* = 0.009) ▪ Median number of new lesions at 2–7 days: 8 (3–16) ▪ Median total new lesion volume: 210 mm^3^ (137–456 mm^3^) ▪ Average new lesion volume: 34 mm^3^ (24–45 mm^3^) ▪ Freedom from brain lesions >150 mm^3^: 87% Freedom from brain lesions >350 mm^3^: 97% ▪ No significant worsening of NIHSS in any of the patients at 30-day follow-up. The largest single lesion volume detected in any patient was 402 mm^3^	This study was a non-randomised, single-arm study that could be considered a remarkable step up from the older ProtEmbo SF study. The results of this study could prove difficult when attempting to compare it with a randomised control group due to lacking a key control group. However, it does provide relatively concrete evidence for the safety and performance of the ProtEmbo device. Furthermore, the comparison with historical TAVI data could have been affected by biasness related to any undesirable baseline and procedural characteristics of patients through the different centres.

MACCE: major adverse cardiac and cerebrovascular events; DW-MRI: diffusion-weighted magnetic resonance imaging; AKI: acute kidney injury; MI: myocardial infarction; TIA: transient ischemic attack; VARC-2: Valve Academic Research Consortium-2; NIHSS: National Institutes of Health Stroke Scale; MoCA: Montreal Cognitive Assessment; mRS: modified Rankin Scale; CPD: cerebral protection device.

## 4. Debates Between Cerebral Protection Devices

Given the presence of these two types of devices and the growing body of evidence supporting their use, it will only be a matter of time before the development of many more CPDs. However, it remains an important concept that will require more randomized controlled trials, preferably larger scale ones across a range of percutaneous transcatheter procedures and patient demographics, to ascertain the better form of embolic protection, i.e., incomplete vs. total full-body embolic protection CPD, and the superior form of CPD, i.e., filter-based capture or deflection devices. There is current evidence that suggests that CPDs offering total full-body embolic protection during percutaneous transcatheter procedures would be significantly more beneficial than those providing incomplete protection. This is particularly so because the left vertebral artery, which branches off the most distant of the aortic arch’s main branches, supplies approximately 20% of the cerebral blood flow [123,124]. During TAVI, the amount and size of embolic debris that passes through the left vertebral artery is observed to be proportionate to the debris amount that passes through the brachiocephalic trunk and left common carotid artery [24,30,102,123]. As such, the idea of total full-body embolic protection CPDs being superior can be inferred simply because it covers more of the vessels that give rise to the brain’s vascular supply hence resulting in better overall perioperative and clinical outcomes. However, as previously mentioned, more studies are needed for this to be finalized. A promising consideration for future research could be assessing the risk of debris size and volume embolization associated with the percutaneous transcatheter procedures prior to deciding between an incomplete or total full-body embolic protection device. The other aspect concerns whether the capture and removal of these embolic debris is superior to deflecting debris away from the brain toward the distal circulation. While it may seem logical for one to assume that capturing debris and removing this material would be advantageous as opposed to dealing with distal complications such as acute renal infarct causing failure and peripheral limb ischemia, it remains to be seen as there is no current evidence to support this belief. As filter-based capture devices are usually larger devices that are inserted typically through femoral access sites, this further increases the risk of vascular complications at the point of insertion and possibly along the vessel [3,8,39].Additionally, the risk-to-benefit ratio specific to procedure type and patient characteristics needs to be carefully considered [23,31]. 

## 5. Cost-Effectiveness of Cerebral Protection Devices

With the rise in adoption of percutaneous transcatheter procedures and the ever-increasing importance of optimizing hospital resources, it remains crucial to determine if CPD usage in these procedures has an evidence-based cost benefit behind them in supporting or rejecting their use across a myriad of settings to aid key healthcare decision making [125]. The risk of clinical neurological events after these transcatheter procedures, albeit low, is not negligible as these neurological events can have drastic impacts on patients’ morbidity and mortality [24,107]. In 2021, stroke was the third leading cause of death globally and was responsible for 7.3 million deaths (10.7% of all deaths) and the fourth leading cause of disability-adjusted life years (DALYs) at 160.5 million DALYs (5.6% of total DALYs). There were 93.8 million prevalent and 11.9 million incident strokes. Ischaemic stroke specifically made up 65.3% of incidents. Stroke burden has been noted to vary by region, country, and socioeconomic status [126]. When it comes to evaluating the true costs associated with strokes, there are multiple components of care that one needs to factor in: loss of productivity, neurorehabilitation, secondary prevention, social services, and resources from informal care. Recent findings suggest that the average per patient per year cost for all identifiable strokes in descending order across certain countries were reported to be USD 232,100 in Australia, USD 59,900 in the United States, Sweden at USD 52,725 followed by Spain at USD 41,950 [126,127]. In the United States, that value increases remarkably to USD 140,000 after factoring in other rehabilitation and long-term care expenditures [128]. In 2017, across Europe, it is estimated that informal care costs amounted to EUR 1.3 billion, cost to healthcare was EUR 27 billion, and the cost due to lost productivity following strokes was EUR 12 billion [129]. In the United States, the indirect costs amounted to 66% of the total costs at USD 103.5 billion, while the cost of productivity loss was USD 38.1 billion and the cost caused by premature death from stroke was USD 30.4 billion [130]. It is worth bearing in mind that these statistics are generic across all forms of strokes, and it is unclear how much of these were associated specifically to the burden of embolic strokes associated with percutaneous transcatheter procedures.

The impact of strokes post-TAVIs are of significant economic concerns as it leads to a 32% increase in cost of index hospitalization, a 121% increase in step down care facility utilization including nursing homes or rehabilitation units, and a 132% increase associated with readmissions—all of which are potentially compounded [131]. With respect to TAVI and CPD usage, a real-world large scale data analysis using data from the National Inpatient Sample and Nationwide Readmissions Database from the period of 2017 to 2018 was conducted for assessing outcomes of CPD use in TAVIs. Approximately 4% of TAVIs in this data pool were conducted with Sentinel. Despite the known benefits of the Sentinel device, multiple logistic regression showed that CPD use was linked to lower adjusted mortality (OR 0.34, 95% CI 0.22–0.52, *p* < 0.01) and fewer neurological complications (OR 0.68, 95% CI 0.54–0.85, *p* < 0.01). Additionally, TAVI with CPD reduced 30-day all-cause readmissions (HR 0.839, 95% CI 0.773–0.911, *p* < 0.01) and stroke (HR 0.727, 95% CI 0.554–0.955, *p* = 0.02). The median costs of hospitalizations were lower when unadjusted (USD 44,880 without CPD vs. USD 44,861 with CPD, *p* < 0.01) and even after propensity matching (USD 44,881 without CPD vs. USD 44,861 with CPD) [132]. Another study that was conducted strategically for data in the last three quarters of 2017 had opposite findings in that TAVI with CPD use was associated with higher median costs (USD 45,050 without CPD vs. USD 47,783 with CPD), even where adjusted (USD 44,578 without CPD vs. USD 47,783 with CPD) [133]. However, these differences could be due to device costs and/or the time in the later part of 2018 when the Sentinel device became approved for reimbursement by the Medicare and Medicaid Services authorities—around the time when utilization increased [28,45,132,134]. Prior to this period, key factors such as the portion reimbursed or the lack of clear clinical evidence were some of the main reasons influencing CPD usage rates. In addition, most TAVI centres currently operate on minimal profit or, worse, are running at a loss [135]. A decision analytic model created to compare the costs and outcomes of the TAVI procedure performed with or without the Sentinel device in the United States found that at 5 years, TAVI with CPD resulted in more quality-adjusted months alive (29.4 vs. 28.7) and a lower cumulative cost per patient (USD 147,711 for CPD vs. USD 148,711 for no CPD). CPD use was more effective and less costly in majority of simulations (57.5%), and in 88.9% of cases, it was cost-effective when considering a threshold of USD 10,000 per quality-adjusted month alive [125]. When it comes to real world studies, multiple studies have demonstrated that Sentinel device use in TAVIs has effectively reduced the rates of strokes, even when performed in patients with AF, and resulted in shorter hospital stays, more routine standard discharges, lower 30-day readmission rates, and improved overall 12-month survival [136,137]. These above findings all show that using CPD during TAVI is economically cost-effective, especially when the Sentinel device is the CPD of choice. On the contrary, a small number of suggestions have also been put forth that consider the already low rate of strokes with TAVI, along with the high cost of the Sentinel device, which costs an approximate USD 3000, and the large treatment ratio—the need to treat 125 patients to prevent one disabling stroke, concluding that it would hence make the cost associated to prevent a single stroke event be about USD 100,000 to USD 154,000. This suggests that the use of the Sentinel device may not be the solution to effectively reduce stroke rates in TAVIs [103,128,131,138]. Overall, a strategy of CPD use across all percutaneous transcatheter procedures for periprocedural stroke remains to be studied in terms of cost-effectiveness and, as such, it is difficult to address if CPDs are indeed cost effective.

Cost effectiveness thresholds are precalculated monetary values to determine if a particular healthcare intervention is cost effective. More recently, two broad approaches have recently been put forth as to how cost-effectiveness thresholds should be estimated [16,139]. The demand-side threshold relates to the willingness to pay for health improvements, and the supply-side threshold reflects the forgone benefits that could have been achieved if the same resources were used in another alternative [140]. This becomes paramount especially in low- and middle-income countries which rely heavily on ensuring that quality healthcare is delivered to meet basic needs within the constraints of their limited resources [140]. In the case of CPD use in low- and middle-income countries, the demand-side threshold would be lacking, if considering current costs effectiveness analyses in the literature, leading to the use of CPDs being somewhat impossible. Though CPDs are currently reimbursed in part or full in certain countries, it would be difficult for low- and middle-income countries to have these systems due to resource constraints. Hypothetically, much needs to be done in terms of lowering the costs of CPDs to justify their use in resource-limited low- and middle-income countries. This could potentially be through optimizing their manufacturing processes, increasing competition in the market, utilizing government or pro bono subsidies, developing more cost-effective materials, and promoting research into next generation designs that are cheaper to produce while not discounting efficacy [125]. Other issues such as a lack of consistent supply chains and catheter laboratory constraints could also make the use of CPDs difficult in these settings. Hence, even though the efficacy of CPDs has been demonstrated, the current primary focus on basic life-saving care and interventions in resource-limited low- and middle-income countries may warrant the adoption of other advanced transcatheter adjuncts, such as CPDs, to be impractical. Furthermore, the ethical considerations that key stakeholders must consider towards using CPDs in resource limited low- and middle-income countries would involve balancing limited access, equitable resource allocation, cultural sensitivity, and, most significantly, the sustainability of these expensive technologies, all while addressing the increasing and pervasive healthcare needs and inequalities in these settings.

## 6. Trajectory of Cerebral Protection Devices into the Future

As the development in the field of CPDs continues to grow in its innovation and adoption to a wider scope of use, multiple trials that could shape the future in maximizing the safety in percutaneous transcatheter procedures within catheter laboratories continue to arise from different parts of the world. One of the most exciting trials currently underway is the BHF PROTECT trial that aims to include an approximate of 7000 TAVI patients and already has a calculated expected 33% relative risk reduction in all stroke events, solely based on a foreseeable 3% stroke prevalence in the control group [98,141]. On completion of the BHF PROTECT trial, its findings could potentially be combined with findings from the PROTECTED TAVR trial to boost statistical significance and to determine the ongoing question of its clinical relevance in TAVI practices. Considering the current lack of evidence of subgroups of patients who could benefit most from TAVI, it remains paramount that studies catered specifically to identifying these groups need to follow closely behind these large trials. This is especially so since TAVI could likely replace surgical aortic valve replacement (SAVR) in the coming future for selected cohort of patients with aortic stenosis. SAVR has stroke rates ranging from 1% to 17%—a wide range that can prove very challenging and costly with post operative cares. In the latest PARTNER 3 trial, it showed that TAVI was superior to SAVR at preventing death, stroke, or rehospitalization at 1 year. These benefits were sustained for 5 years. Mortality and strokes at 5 years were similar between the two modalities. TAVI was also associated with a lower incidence of atrial fibrillation, and a shorter hospital length of stay compared with SAVR. TAVI was also associated with a larger improvement in quality of life compared with SAVR upfront. A notable finding was a non-statistically significant numerical increase in the need for new permanent pacemakers within 30 days in the TAVI group (6.5% with TAVI, 4.0% with SAVR) [142]. A propensity-matched study conducted on 1204 pairs of patients with severe aortic stenosis undergoing TAVI or SAVR in the previous PARTNER trial found similar findings of a greater 30-day stroke incidence after SAVR compared with TAVI (3.9% vs. 2.2%, *p* = 0.018) [143]. However, for patients where TAVIs are not possible, a previous study trialed two unnamed CPD devices, one suction-based and one filtration-based, that were not able to prevent clinical strokes or CELs [31]. Hence, there remains room for current CPDs to be studied in a SAVR setting. Other upcoming trials for full body protection filter-based capture devices also include the EMBOLINER IDE and EMBLOK trials. While the upcoming deflector CPD trials include the PROTEMBO IDE and GUARDIAN trials, all remain in the early stages. These could eventually produce key data informing primarily of devices’ safety and efficacy. Once again, cost-effective studies should also be considered paramount when large scale data become available on other CPDs to allow for the holistic consideration of the adoption of these devices across an international platform. The newer generation devices are all seemingly being designed to correct the limitations of previous CPDs, such as incomplete aortic arch protection or distal embolism risks and inadaptability for use with current TAVI procedural systems [141].

Designing clinical trials for CPDs for percutaneous transcatheter procedures presents a unique set of challenges, particularly with meaningful endpoint selections. Traditionally, stroke outcomes are rare but not negligible, and this would mean that a large sample size is needed to detect differences. This has been previously highlighted across the different studies’ limitations in Table 2. This has thus greatly limited many trials due to low statistical power. Many CPD studies have instead adopted an imaging-based surrogate endpoint, like with the use of DW-MRI for detecting CELs. To gain statistical traction, larger populations and populations with an array of comorbidities are needed. However, this would undeniably introduce different confounding factors complicating trials, thus increasing the requirements for research finances and resources. Researching and determining appropriate follow-up duration post-TAVIs would also prove beneficial to ensuring trials are conducted uniformly and fairly to allow researchers and/or clinicians to draw comparative conclusions across studies. In addition, the use of validated cognitive assessment tools uniformly across future studies to evaluate efficacies are also key. Standardizing endpoints, such as those outlined by the VARC-3, will improve consistency across studies. In conclusion, larger, well-powered trials are needed to draw concrete stroke rates across devices and procedural types, and it is also especially important to incorporate a broader array of population baselines, or alternatively, focusing solely on either low or high-risk groups. Uniformity across long-term follow-up with a cognitive assessment tool being used, patient-reported outcomes, and the evaluation of patient perspectives to enable accurate applicability should be a standard. Additionally, direct comparisons of different CPDs are paramount to determining which systems offer the best protection. Lastly, cost-effectiveness analyses should be integrated into clinical trials or have dedicated studies for exploring cost-effectiveness especially in low-resource settings to ensure that the benefits of CPDs are reaped internationally, even where healthcare resources can be scarce and to justify their use in daily practice across percutaneous transcatheter procedures.

## 7. Conclusions

Stroke complicating cardiac percutaneous transcatheter procedures remain largely unpredictable and pose major issues of clinical concern for clinicians and the loved ones of patients receiving these procedures. Despite the advancements in technology and operator skills, the risk has not been significantly negated. Given the rising numbers and array of cardiac procedures performed percutaneously and with most of these strokes being related to embolization during these procedures, the role of CPDs has never been more important than in current times. Their applications have been shown to provide added benefits to patient safety across the many studies available. A large proportion of current evidence encompassing CPD use exists in TAVI patients, and a large proportion of these are of the Sentinel CPD as TAVI remains the transcatheter cardiac procedure with the highest procedure-related stroke. There remain greatly limited data surrounding CPD use in other percutaneous transcatheter procedures performed in cardiac catheterization laboratories, and as such, we do not have an evidence-based consensus for its routine use. Overall, the rise of CPDs is anticipated and welcomed by many in the field of percutaneous transcatheter procedures within cardiac catheterization laboratories; however, more work is needed to allow for the definitive establishment of the potential that CPDs can provide.

## Data Availability

Not applicable.
